# Endothelial cells and macrophages as allies in the healthy and diseased brain

**DOI:** 10.1007/s00401-024-02695-0

**Published:** 2024-02-12

**Authors:** Adam Denes, Cathrin E. Hansen, Uemit Oezorhan, Sara Figuerola, Helga E. de Vries, Lydia Sorokin, Anna M. Planas, Britta Engelhardt, Markus Schwaninger

**Affiliations:** 1 “Momentum” Laboratory of Neuroimmunology, Institute of Experimental Medicine, Budapest, Hungary; 2 Department of Molecular Cell Biology and Immunology, Amsterdam UMC Location Vrije Universiteit Amsterdam, Amsterdam, The Netherlands; 3 Amsterdam Neuroscience, Amsterdam UMC, Amsterdam, The Netherlands; 4 MS Center Amsterdam, Amsterdam UMC Location VU Medical Center, Amsterdam, The Netherlands; 5 Institute of Experimental and Clinical Pharmacology and Toxicology, Center of Brain, Behavior and Metabolism, University of Lübeck, Lübeck, Germany; 6 Department of Neuroscience and Experimental Therapeutics, Instituto de Investigaciones Biomedicas de Barcelona (IIBB), Consejo Superior de Investigaciones Cientificas (CSIC), 08036 Barcelona, Spain; 7 Cerebrovascular Research Group, Institut d‘Investigacions Biomediques August Pi I Sunyer (IDIBAPS), Barcelona, Spain; 8 Faculty of Medicine, University of Barcelona, Barcelona, Spain; 9 Institute of Physiological Chemistry and Pathobiochemistry, University of Muenster, Munster, Germany; 10 Cells-in-Motion Interfaculty Centre (CIMIC), University of Münster, Münster, Germany; 11 Theodor Kocher Institute, University of Bern, Bern, Switzerland; 12 German Research Centre for Cardiovascular Research (DZHK), Partner Site Hamburg, Lübeck, Kiel, Germany

**Keywords:** Microglia, Endothelial cells, Perivascular macrophage, Angiogenesis, Vascular remodelling, Neuroinflammation, Stroke, Multiple sclerosis, Alzheimer’s disease, Antigen presentation

## Abstract

Diseases of the central nervous system (CNS) are often associated with vascular disturbances or inflammation and frequently both. Consequently, endothelial cells and macrophages are key cellular players that mediate pathology in many CNS diseases. Macrophages in the brain consist of the CNS-associated macrophages (CAMs) [also referred to as border-associated macrophages (BAMs)] and microglia, both of which are close neighbours or even form direct contacts with endothelial cells in microvessels. Recent progress has revealed that different macrophage populations in the CNS and a subset of brain endothelial cells are derived from the same erythromyeloid progenitor cells. Macrophages and endothelial cells share several common features in their life cycle—from invasion into the CNS early during embryonic development and proliferation in the CNS, to their demise. In adults, microglia and CAMs have been implicated in regulating the patency and diameter of vessels, blood flow, the tightness of the blood–brain barrier, the removal of vascular calcification, and the life-time of brain endothelial cells. Conversely, CNS endothelial cells may affect the polarization and activation state of myeloid populations. The molecular mechanisms governing the *pas de deux* of brain macrophages and endothelial cells are beginning to be deciphered and will be reviewed here.

## Introduction

Brain endothelial cells form the blood–brain barrier (BBB) and play a key role in providing the special homeostatic internal milieu required for optimal functioning of central nervous system (CNS) neurons. To accomplish this, endothelial cells cooperate with mural cells such as pericytes and smooth muscle cells, with cell types of the CNS parenchyma and, importantly, with CNS resident myeloid populations; together, they form the neurovascular unit [109]. As immune cells, resident myeloid cells are responsive to changes in the CNS parenchyma that occur during tissue injury and inflammation or during normal development and adaption to a changing environment. Their shared roles in maintaining tissue homeostasis in the CNS make macrophages and endothelial cells natural allies. Previous reviews have comprehensively covered the interactions of endothelial cells and macrophages [24, 119], but recent progress, in particular on microglial-endothelial interactions, makes an update worthwhile.

## Diversity of endothelial cells and CNS macrophages

Both endothelial cells and macrophages in the CNS are heterogeneous cell populations. This concept has been underlined by recent single-cell and single-nuclei RNA sequencing (sc/nRNAseq) studies. Most microvascular endothelial cells in the CNS are non-fenestrated and form a tight BBB. Fenestrated, Plasmalemma Vesicle-Associated Protein (PLVAP)-positive microvascular endothelial cells are found in the mature brain only in circumventricular organs (Table 1). Recent sc/nRNAseq studies have supported the view that brain endothelial cells differ in different zones of the vascular tree [56, 58, 121]. Arterial, capillary, and venous endothelial cells are distinguishable by characteristic transcriptomic profiles (Table 1). Arterial endothelial cells are rich in transcription factors, capillary endothelial cells express transporters such as Major Facilitator Superfamily Domain-containing protein 2A (MFSD2a) at high levels and subsets of venous endothelial cells express adhesion molecules required to support leukocyte adhesion and extravasation or genes allowing proliferation [56, 58, 121]. Beyond differences between vascular zones, it is apparent that endothelial cells in different parts of the brain possess area-specific properties, e.g., forebrain endothelial cells differ from hindbrain endothelial cells [10, 56, 125]. However, the functional consequences of this diversity for endothelial–macrophage interactions are poorly understood.

**Table 1:** Genetic markers associated with the different myeloid cell subsets and endothelial cells in the CNS

Endothelial Cells	Genes
Fenestrated	Plvap [39], Gpihbp1 [39], Plpp3 [39]
Arterial	Bmx [36, 39, 58, 121], Fbln5 [58], Gkn3 [36, 39, 58, 121], Igfbp4 [58], Mgp [36, 39, 121], Stmn2 [36, 39, 58], Vegfc [10, 36, 121], Alpl [10, 58], Cxcl12 [36, 58], Efnb2 [10, 36, 121], Sema3g [36, 121]
Capillary	Angpt2 [10], Ivns1abp [121], Mfsd2a [10, 121], Slc7a5 [10, 121], Rgcc [36]
Capillary arterial	Alpl [10, 58], Cxcl12 [36, 58], Efnb2 [10, 36, 121], Glul [36], Sema3g [36, 121], Tgfb2 [121]
Capillary venous	Abcg1 [58], Car4 [36, 39], Map7 [58], Slc16a1 [10, 36, 121], Tfrc [10, 39, 58, 121]
Venous	Adh1 [58], Icam1 [39, 58], Lcn2 [36, 39], Nr2f2 [36, 58, 121], Slc38a5 [36, 39, 121], Tmsb10 [36, 58]
Large vessel	Vcam1 [36, 39, 121], Vwf [36, 58, 121]
**Brain macrophages**	**Genes**
Microglia	Tmem119 [39, 58, 60], P2ry12 [36, 39, 58, 60], Hexb [36, 39, 58, 75], Olfml3 [39, 58], Siglech [58, 60]
CNS-associated macrophages	Mrc1 [36, 39, 58, 60], Tgfbi [36, 58] Lyve1 [58, 60], Cd163 [36, 60, 92], Siglec1 [60, 92], Plf4 [60], Ms4a7 [60]
Monocyte-derived cells	Ly6c2 [39, 60], Ccr2 [39, 60, 104], Plac8 [60], Anxa8 [60], Nr4a1 [60]

**Fig. 1:**
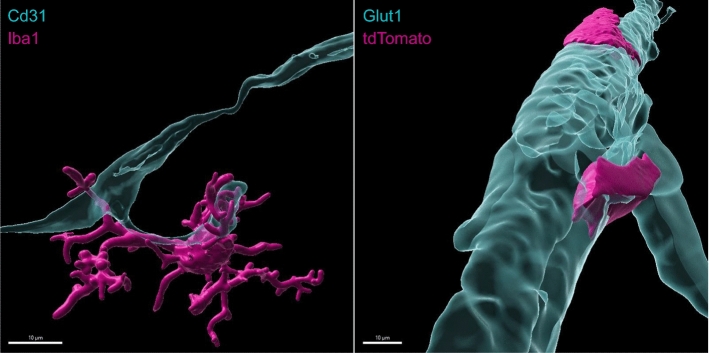
3D reconstructions show that Cd31- or Glut1-positive endothelial cells are closely adjacent to Iba1-positive microglia (left) and tdTomato-positive perivascular macrophages (right)

An important aspect that has emerged over the last years is the distinction of CNS compartments with different macrophage populations, namely the parenchyma and CNS borders, including meninges, choroid plexus, and perivascular spaces. CNS compartments are populated by a variety of tissue-resident macrophages, including parenchymal microglia and extra-parenchymal CNS (or border)-associated macrophages (CAMs). The latter are located at selective CNS barriers within the perivascular space (perivascular macrophages, PvMΦ), the choroid plexus (cpMΦ), and in the subarachnoid space bordered by the leptomeninges (leptomeningeal macrophages, MnMΦ)[2]. Microglia and CAMs originate from common prenatal erythromyeloid progenitors, but then follow different differentiation paths during the development of the CNS, with locations of CAMs offering a *niche* for their cellular specification, as shown using rodent models [74]. The development of microglia and CAMs is reviewed in a parallel article [20]. Specific characteristics of PvMΦ depend on their location adjacent to arterioles and venules, not capillaries (Fig. 1)[30]. They are sandwiched between the glia limitans and the outer aspect of the vascular wall so that they acquire an elongated shape. In addition to location, PvMΦ and microglia can be distinguished by a few specific markers (Table 1). Mouse and human CAMs share MRC1, F13A1, STAB1, and CD163 as markers, while P2Y12R, SLC2A5, SELPLG, and SPP1 are conserved in humans and mice as microglial markers [103]. The activation state of microglia is reflected by morphological changes and by the expression of markers such as CD68, SPP1, and IGF1 [48]. Based on sc/nRNAseq analyses, genome-wide association, or morphological studies, different names for subsets of microglia have been proposed, such as “disease-associated microglia”, “injury-activated microglia”, or “perivascular microglia” but there is no consensus on their definition or standardized nomenclature [88]. Rather, it is apparent that the function of given microglial phenotypes is highly context- and disease-dependent.

In adulthood, when the vascular barriers are established, myeloid cells do not cross the endothelial monolayer and underlying basement membrane, except during inflammation. However, in neuroinflammatory diseases, monocytes are recruited to the CNS. The recruitment depends on adhesion molecules and other proteins on the luminal side of the vessel that control the multi-step extravasation of monocytes across the endothelial monolayer. In experimental autoimmune encephalomyelitis (EAE), a model of multiple sclerosis (MS), scRNAseq experiments have suggested that monocytes differentiate into several macrophage subtypes that localize to the choroid plexus and the leptomeninges, less so in the perivascular space [60]. In a model of ischemic stroke, inflammatory monocytes differentiated into two subtypes of monocyte-derived macrophages expressing a mainly neuroprotective gene set [39]. Importantly, monocyte-derived macrophages can be distinguished from CNS resident macrophages based on their transcriptome profile (Table 1).

## Endothelial cells and microglia are in direct contact

From early development to adulthood, microglia and endothelial cells interact. Microglia originate from primitive yolk sac progenitors, the erythromyeloid progenitors, and colonize the developing CNS around E9.5 in mice and after the 4.5th gestational week in humans (reviewed in [20]). Interestingly, the erythromyeloid progenitors not only give rise to microglia but have been shown to provide a complementary source of endothelial cells that are recruited to a pre-existing vasculature [96].

Although microglial cells are associated with the CNS vasculature from early developmental stages up to adulthood, the molecular anatomy of microglia–vascular interactions, their function and changes in different brain diseases have not been extensively studied until recently. In the adult brain, microglia establish an intimate association with the vasculature (Fig. 1). These associations are apparent in the arterial, capillary, and venous zones, although the differences in the vascular area contacted and specific cell–cell interactions established remain to be precisely defined in different vascular beds. While microglial cell bodies reside in the brain parenchyma (mostly isolated from endothelial cells by both endothelial and astroglial basement membranes), a subset of microglial cell bodies is vessel-associated. However, even when the cell soma is not associated with blood vessels, virtually, all microglia send some processes to the vicinity of endothelial cells along the vascular tree. In fact, recent research demonstrated that microglia establish direct cell–cell contacts with all cells of the neurovascular unit, including endothelial cells, pericytes, smooth muscle cells, astrocytes, and neurons as well as PvMΦ, as confirmed by high-resolution confocal microscopy, super-resolution microscopy, immunoelectron microscopy, and electron tomography [8, 17, 139].

Immunogold labelling of P2Y12R, a microglia-specific marker in the brain parenchyma, and extensive electron microscopic analyses showed that microglial processes can extend beyond the perivascular astroglial endfeet and contact endothelial cells directly at sites devoid of astroglial coverage in both the mouse and the human brain [17]. Moreover, elevated levels of P2Y12R in microglial processes contacting endothelial cells in the vicinity of endothelial mitochondria have been demonstrated, resembling purinergic junctions between microglial processes and neuronal cell bodies at sites of neuronal ATP release [17, 18]. Of note, 3D reconstructions demonstrated that processes of individual microglia directly contact several neurons, astrocytes, and endothelial cells [17, 18, 116], illustrating that they are optimally positioned to influence complex intercellular interactions in the neurovascular unit, such as astrocyte-mediated changes in vascular tone or neurovascular coupling. In vivo two-photon imaging studies revealed that microglia–vascular contacts are highly dynamic under physiological conditions and are strongly influenced by the regulation of vascular diameters [7, 17]. Contacts of microglial processes have an average life-time of 5–15 min, which may differ substantially in different vascular beds. The molecular composition and precise function of microglial contacts with other cell types within the neurovascular unit remain to be defined. Similarly, numerous mechanisms, including those mediated by P2Y12R, PANX1 channels, TGFβ1, vascular endothelial growth factor (VEGF), CX3CL1/CX3CR1, CXCR4/CXCL12, IL-34/CSF1R, CCL5/CCR5, and other molecular pathways have been documented to contribute to microglia–vascular interactions, but the function of these pathways in the formation of physical interactions between microglial cell bodies, processes, and filopodia with cells of the neurovascular unit is presently unclear [17, 23, 35, 52, 81, 139]. Nevertheless, accumulating data show that the dysfunction of microglia–vascular interactions is associated with broad vascular alterations in different forms of CNS injury or disease.

## Macrophages modulate cerebral blood flow (CBF) and hypoperfusion

**Fig. 2:**
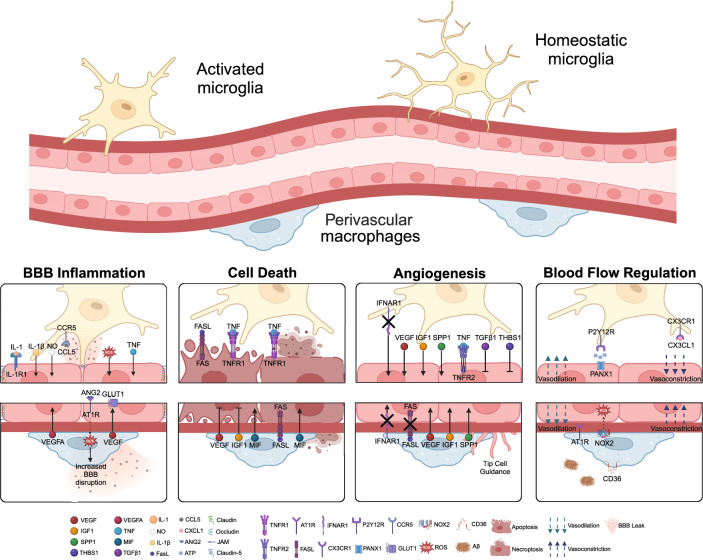
Scheme summarizing interactions between microglia (top) and PvMΦ (bottom) and brain endothelial cells in microvessels. Microglia or PvMΦ modulate angiogenesis, endothelial cell death, and barrier properties of brain endothelial cells. In addition, microglia and PvMΦ interact with endothelial cells to regulate blood flow. As representative microvessel, we have depicted an arteriole, but some of the effects were observed in capillaries or venules as specified in the text. Dark red line, endothelial and astroglial basement membranes. Ang2, angiotensin 2. AT1R, angiotensin 2 receptor 1. BBB, blood–brain barrier. CCL5, C–C motif chemokine ligand 5. CX3CL1, fractalkine. CX3CR1, fractalkine receptor. FASL, FAS ligand. GLUT1, glucose transporter 1. IFNAR1, interferon α/β receptor 1. IGF1, insulin-like growth factor 1. IL-1, interleukin-1. IL-1R1, IL-1 receptor 1. PANX1, pannexin 1. ROS, reactive oxygen species. TNF, tumour necrosis factor. TNFR1/2, TNF receptor 1/2. VEGF, vascular endothelial growth factor

Selective manipulation of microglia or CAMs has recently become technically feasible. Pharmacological inhibitors of colony-stimulating factor 1 receptor (CSF1R), such as PLX5622 or PLX3397, are suitable for the rapid depletion of microglia and CAMs in vivo [2]. This regimen induces apoptotic cell death limiting the release of inflammatory mediators into the brain [27, 116]. As CSF1R is also expressed outside of the brain, additional effects on bone marrow-derived macrophages, other tissue macrophages and subsets of dendritic cells should be taken into consideration when interpreting experiments [2, 65, 114]. The intracerebroventricular injection of clodronate liposomes affords the selective deletion of PvMΦ and MnMΦ [2]. Importantly, induction of macrophage cell death can stimulate the release of pro-inflammatory cytokines as a confounding factor [2]. Therefore, it is suggested to also use alternative approaches (i.e., pharmacological blockade of specific receptors on microglia or CAMs and genetic interventions) to validate the results of cell deletion. For cell-specific conditional gene deletion, there are several Cre driver mouse lines available [25]. Recently, the Mrc1CreER^T2^ line has been added to the arsenal, which allows cell-specific gene deletions in CAMs [108]. Using both pharmacological and genetic tools, recent data suggest that microglia contribute to CBF, hypercapnia-induced vasodilation and neurovascular coupling via purinergic actions, which are partially additive to the effects of nitric oxide (NO) and may involve adenosine [17]. In vivo two-photon imaging studies showed that intracellular calcium changes in microglia and the recruitment of microglial processes or filopodia to arterioles occur within 1–2 min after hypercapnia, suggesting that microglia can respond to vascular changes rapidly and may participate in CBF modulation or vascular adaptation even under physiological conditions. Supporting this, baseline CBF was increased after microglia depletion by PLX3397 and in P2Y12R- or PANX1-deficient mice, suggesting that microglia may be implicated in the regulation of cerebrovascular tone (Fig. 2) [7]. Conversely, vasodilation in response to hypercapnia was impaired upon microglia depletion and in P2Y12R- and PANX1-deficient mice [7, 17]. It should be noted that P2Y12R is specific for microglia in the brain and is not expressed by CAMs under homeostatic conditions (Table 1). Purinergic mechanisms of microglia-vascular interactions are likely to operate in parallel with several other modulatory pathways. For example, activation of microglial fractalkine (CX3CL1)–CX3CR1 signalling led to capillary constriction, while blocking the renin–angiotensin system with candesartan abolished microglia-mediated vasoconstriction in the mouse retina (Fig. 2) (Table 2) [81].

**Table 2:** Overview of factors expressed or released by different macrophage populations that act on endothelial cells

Factor	Expressed by	Vascular effects	Model	Reference
Angiotensin II	Microglia	Capillary constriction	Streptozotocin-induced diabetic retinopathy	[81]
FASL	Macrophages	Endothelial cell death	Inhibition of miR-30a in mouse retina	[83]
IGF1	Microglia	Angiogenesis	Mouse model of oxygen-induced proliferative retinopathy, crush-injured spinal cord in neonatal mice	[69] [66]
IL-1β	Microglia	Vascular activity changes mediated by endothelial IL1R1	Mouse model of LPS preconditioning in ischemic spinal cord injury	[37, 84]
MIF	Monocyte-derived macrophages	Endothelial necroptosis and apoptosis	Mouse model of perioperative ischemic stroke	[67]
ROS	Microglia	Tight junction disruption	In vitro	[115]
	PvMΦ	BBB disruption Impairment of endothelium-dependent vasodilation	Mouse model of chronic hypertension Cerebral blood flow in the mouse cortex	[107] [90]
TGFβ1	Microglia	Modulation of vascular architecture, modulation of endothelial proliferation	Retina development; in vitro	[23, 124]
TNF	Microglia	Enhanced endothelial proliferation (TNFR2); endothelial necroptosis (TNFR1); tight junction disruption	In vitro	[124] [14] [115]
VEGF-A	PvMΦ	Induction of endothelial GLUT1 expression	High-fat diet	[57]
	Microglia		Cerebrovascular injury	[73]
	CAMs	BBB disruption	MCAO, hypoxia	[92, 110]

*FASL* FAS ligand, *IGF1* insulin-like growth factor 1, *MCAO* middle cerebral artery occlusion, *MIF* macrophage migration inhibitory factor, *ROS* reactive oxygen species, *TGFβ1* transforming growth factor β1, *TNF* tumour necrosis factor, *VEGF* vascular endothelial growth factor

Based on these findings, it is likely that phenotypic changes in microglia and their altered interactions with other cells of the neurovascular unit observed in pathological conditions, such as during acute or chronic hypoperfusion, cerebral ischemia or haemorrhage, contribute to the associated changes in blood flow. This is supported by the altered cerebrovascular reactivity and impaired adaptation to cortical hypoperfusion observed in the absence of microglia through PLX5622 treatment or blocking of P2Y12R [17]. Interestingly, impaired vascular adaptation to hypoperfusion induced by repeated, unilateral common carotid artery occlusion was also apparent in the contralateral hemisphere, arguing for widespread effects of microglial modulatory actions on apparently large vascular territories [17]. However, the role of these mechanisms in the regulation of vascular tone and perfusion changes in vascular beds remains to be defined.

It is likely that microglia located close to brain microvessels have unique regulatory activities [7, 17], which still require elucidation. Of note, microglial activity changes rapidly in cerebral ischemia or vascular injury and precedes BBB damage. For example, changes in microglial morphology and process dynamics were associated with capillary CBF changes during bilateral common carotid artery occlusion [76] and microglia formed clusters around blood vessels even before BBB injury was detected [116]. Repeated unilateral common carotid artery occlusion also rapidly increased the motility of microglial processes [17]. Given that inflammatory actions on cerebral endothelial cells contribute to perfusion deficits after cerebral ischemia via IL-1R1 [127], it is interesting to note that systemic inflammatory stimuli also markedly alter microglial morphology and lead to ensheathment of blood vessels by microglial processes [131]. However, the contribution of inflammation-related microglial phenotype changes to CBF in different disorders remains to be defined. The fact that microglia-mediated processes take place at different temporal and spatial scales argues for the need for extensive in vivo imaging studies combined with the use of biosensors and cell-specific labelling to understand the underlying mechanisms. In the future, proteomics, metabolomics, and other approaches could be used to identify the mediators that influence microglial responses and cell–cell interactions in the neurovascular unit under different physiological and pathological conditions. This could clarify how microglia influence the action of different vasoactive metabolites involved in CBF regulation. In addition to microglia, CAM have also recently been implicated in the regulation of blood flow. As discussed below [‘Microglia and perivascular macrophages (PvMΦ) control BBB permeability and neuroinflammation’], PvMΦ have been associated with the development of neurovascular and cognitive dysfunction in hypertension [31]. PvMΦ depletion, using clodronate liposomes, ameliorated CBF response to whisker–barrel cortex stimulation by acetylcholine in chronically hypertensive BPH/2J mice [31]. In a mouse model of Alzheimer’s disease (AD), the depletion of PvMΦ and possibly other CAMs by clodronate liposome treatment reduced vascular oxidative stress [90]. The latter was mediated by CD36 and NOX2 in CAMs and was associated with impaired neurovascular coupling and cognitive function [90, 120].

These results collectively suggest that myeloid populations in the CNS may exert complex and heterogeneous effects on different compartments. Understanding the underlying mechanisms may have considerable impact on the development of novel diagnostic and therapeutic tools in brain disorders.

## Microglia and perivascular macrophages (PvMΦ) induce angiogenesis

The development of microglia and blood vessels in the brain is closely connected [20]. During development and into adulthood, microglia regulate angiogenesis, a concept largely based on studies in the retina. Pharmacological depletion of microglia by intravitreal injections of clodronate liposomes reduced the vessel density in the retina of new-born rats [13]. Conversely, activation of microglia and macrophages by the deletion of the interferon α/β receptor (IFNAR) in CX3CR1-positive resident cells enhanced angiogenesis in the mouse retina (Fig. 2) [71]. Also, in the brain, loss of microglia due to the deficiency of PU.1 and CSF1 led to a decreased vascular complexity in mice [29].

Macrophages influence brain angiogenesis at different levels. Live imaging in zebrafish suggested that microglia help nascent sprouts to form anastomoses [29]. In addition, brain macrophages can stimulate sprouting of tip cells by expressing pro-angiogenic growth factors. Interestingly, growth factors expressed by macrophages differ between experimental paradigms and CNS regions: VEGF expression in PvMΦ was reported to be upregulated by high-fat diet [57] and in microglia by cerebrovascular injury [73] (Table 2). By contrast, in a mouse model of oxygen-induced proliferative retinopathy, retinal microglia did not express increased levels of VEGF; rather, IGF1 was upregulated and stimulated angiogenesis (Fig. 2) [69]. scRNAseq showed that IGF1 and SPP1 were expressed by microglia exhibiting a high glycolytic transcriptomic signature. The latter two genes are also highly expressed in an activated microglia subtype prevalent during early postnatal development [48] and have been proposed to be potential mediators of angiogenesis in a rat spinal cord injury model [133]. An activated microglia subtype expressing IGF1 and other genes promoting angiogenesis has been linked to a scar-free repair of the crush-injured spinal cord in neonatal mice [66]. Interestingly, this subtype of microglia in the neonatal mouse spinal cord expresses not only pro-angiogenic genes but also the anti-angiogenic thrombospondin-1 (Thbs1) (Fig. 2). In vitro*,* microglia have been reported to either stimulate or inhibit endothelial cell proliferation, depending upon the microglial activation state [124]. Homeostatic microglia released TGFβ1 inhibiting endothelial proliferation, while LPS-activated microglia secreted TNF that enhanced proliferation, possibly by TNFR2-mediated processes (Fig. 2) [124]. In vivo, genetic engineering can induce anti-angiogenic activity of microglial cell lines [72], but the role of endogenous microglia as potential inhibitors of angiogenesis is still largely unexplored.

## Macrophages impact the viability of endothelial cells in the CNS

Since macrophages stimulate the formation of new blood vessels, it seems plausible that they also support their survival. Indeed, macrophages express genes, such as VEGF or IGF1, that are known to act as survival factors and inhibit endothelial apoptosis induced by hyperoxia, cytokines or oxidative stress, in addition to their function as stimuli of angiogenesis [1, 50]. However, it is still unclear whether macrophages prevent endothelial cell death in cerebrovascular disease by releasing growth factors [141]. Although the underlying mechanisms are unknown, there is evidence that macrophages protect endothelial cells in models of cerebrovascular damage. In a mouse retinal vein branch occlusion model, monocyte-derived macrophages reduced apoptotic death of endothelial cells [104]. Moreover, in a neonatal rat stroke model, microglial depletion by intracerebral administration of clodronate liposomes (> 85% reduction) exacerbated vessel rarefaction and endothelial cell death, detected by immunostaining of active-caspase 3-positive endothelial cells and the appearance of string vessels [32]. The latter are empty basement membrane tubes that remain after the death of endothelial cells. Without protection by microglia, the aggravated endothelial cell death in this study led to BBB disruption and intracerebral haemorrhage [32]. By contrast, in neuroinflammatory diseases, activated macrophages release several cytokines that can induce endothelial cell death. One such cytokine is macrophage migration inhibitory factor (MIF), which was shown to be elevated on circulating myeloid cells in patients after surgery [67] and in mice subjected to peripheral surgery followed by middle cerebral artery occlusion (MCAO), a model of perioperative ischemic stroke (Table 2). In this experimental paradigm, MIF-expressing macrophages, which were probably monocyte-derived macrophages, occurred close to blood vessels in the ischemic hemisphere [67]. While MIF had no effect on blood vessels under normal conditions, it induced necroptotic and apoptotic cell death of endothelial cells after ischemia (Fig. 2). The MIF-induced damage of capillaries disrupted the BBB and increased both infarct size and neurological deficit of mice.

TNF is another factor released by macrophages that damages endothelial cells, at least in in vitro ischemia of cerebral endothelial cells (Table 2). Being induced in ischemic microglia through NF-κB signalling, TNF induces necroptosis in endothelial cells by binding to the TNF receptor 1 (TNFR1) (Fig. 2) [14]. Importantly, endothelial cell death could be mitigated by the anti-TNF antibody, infliximab. Interestingly, TNF has also been reported to stimulate angiogenesis [124]. The reasons for these opposing effects of TNF may be due to the receptor profile of different endothelial cell types or endothelial cells at different developmental stages, with prevalence of TNFR1 having pro-apoptotic effects and prevalence of TNFR2 being pro-angiogenic (Fig. 2) [124]. A third cytokine that has been implicated in the macrophage-induced death of endothelial cells is FASL (CD95L). Macrophage/microglia-derived FASL can bind to the endothelial receptor FAS (CD95), that contains a death domain, to trigger cell death but is normally inhibited by miR-30a [83]. Inhibition of miR-30a in the mouse retina increased endothelial FAS levels, triggering endothelial cell death [83]. However, normal FAS expression does not seem to be sufficient to mediate the killing of endothelial cells by microglial FASL, because deleting FAS in endothelial cells did not affect endothelial cell death in the retina or cortex of early postnatal mice, although it did stimulate angiogenesis [15]. In the ischemic brain, microglia can phagocytose endothelial cells, a process that has been suggested to contribute to the disruption of the BBB [59]. In summary, it appears that different CNS myeloid populations can enable the plasticity of vessels by promoting angiogenesis, support the survival of endothelial cells under stress, and stimulate the death and removal of damaged endothelial cells in different scenarios.

## Perivascular macrophages (PvMΦ) and vessel remodelling

In extracranial vessels, PvMΦ have been implicated in the long-term physiological remodelling of rodent carotid and mesenteric arteries, where high expression of fibrillar collagens characteristically occurs and, in the case of mesenteric arteries, affects their ability to either expand or contract in response to changes in blood flow [86]. In retinal blood vessels, PvMΦ have been reported to occur in regions of the basement membrane characterized by low levels of collagen IV [80]. These studies raise the possibility that PvMΦ affect the expression of extracellular matrix (ECM) molecules by adjacent vascular wall-constituting cells, probably through the secretion of cytokines as recently suggested by a study on peritoneal macrophages [138]. Apart from contributing to the barrier function of the vascular endothelial basement membrane, ECM components have the capability to bind and present cytokines and chemokines [112], and macrophages can produce several proteases that can selectively cleave ECM molecules or their receptors, as well as cytokines and chemokines trapped in the ECM [reviewed in 49].

A recent acute ischemic stroke study in which PvMΦ were deleted using clodronate liposomes has also suggested that PvMΦ induce changes in the ECM. Transcriptomic analysis of isolated CD163-positive myeloid populations from rat brains 24 h after transient cerebral ischemia has revealed changes in gene expression profiles [92]. Among the top 25 genes that showed increased expression were genes associated with the ECM, including versican, syndecan, fibronectin-1, SPP1, as well as semaphorin-3c and the matrix metalloproteinases (MMPs) MMP7 and MMP14. Enriched biological pathways in the CD163-enriched population after ischemia included ECM, extracellular space, MMPs, collagen degradation, and blood vessel remodelling [92, 97]. These features suggest a role of PvMΦ in scavenging and removing components of damaged basement membranes or CNS parenchymal matrix after stroke. Although MMPs produced by PvMΦ could potentially play a role in ECM cleavage following brain injury, the current studies suggest that this is not their main function in vivo; rather, they may also be involved in angiogenesis and neurovascular remodelling through the modulation of cytokine and/or chemokines and their receptors. Notably, a recent study indicates that PvMΦ influence the remodelling of the ECM, impacting arterial movement, which emerges as a significant contributor to cerebrospinal fluid (CSF) flow dynamics [22]. Employing pharmacological or genetic approaches to diminish PvMΦ correlated with an accumulation of ECM proteins, obstructing fluid passage in perivascular spaces and ultimately impairing fluid perfusion and clearance [22]. Moreover, the pharmacological stimulation of PvMΦ alleviated age-related dysfunction in these cells and reinstated normal CSF dynamics [22]. These findings reinforce that PvMΦ may serve as a potential source of molecules that affect remodelling of the ECM and regulate brain waste clearance. The molecular mechanisms of these effects, however, remain to be elucidated.

Chronic impairment of the brain fluid flow may have implications for neurodegenerative diseases such as AD and chronic SVD, including sporadic SVD, monogenic SVD, and cerebral amyloid angiopathy (CAA). Indeed, it has been suggested that brain fluid flow is altered in a mouse model of AD and that this impairment may contribute to Aβ accumulation [93]. However, the invasive methods employed and resulting changes in CSF pressure may have impacted on these results. Dysfunction of the brain fluid flow could also play a role in sporadic SVD. A typical feature of cerebral SVD is the presence of enlarged perivascular spaces. In this pathology, it has been proposed that enlarged perivascular spaces are the manifestation of impaired clearance of waste proteins from the interstitial fluid space [9]. Altered waste clearance could be involved in genetic forms of SVD, such as cerebral autosomal dominant arteriopathy with subcortical infarcts and leukoencephalopathy (CADASIL), caused by mutations in the NOTCH3 gene. CADASIL is characterized by penetrating artery dysfunction leading to reduced blood flow, cerebrovascular reactivity, and increased BBB leakage. The latter is due to small brain vessel injury associated with the accumulation of granular osmiophilic material, which contains the extracellular domain of NOTCH3 and ECM proteins [16]. Pericyte loss around brain capillaries has been reported to result in the detachment of astrocytic endfeet at blood vessel borders and the disruption of the perivascular space in a mouse model of CADASIL [40]. Notably, perivascular enlargements in CADASIL are associated with an inflammatory cell response around the blood vessels, leading to an increase in the number of PvMΦ, as detected in brain tissue samples from CADASIL patients [87].

## Phagocytosis by perivascular macrophages (PvMΦ)

The phagocytotic capacity of PvMΦ is evident in blood vessels of the area postrema and other circumventricular organs where microvascular endothelial cells lack tight junctions and, consequently, do not form a tight barrier for the soluble factors in the blood. The large perivascular spaces within the area postrema show increased numbers of PvMΦ, which express CD169 (Siglec1) and/or the scavenger receptor CD163 [126]. PvMΦ have been reported to associate with endothelial junctions [80], where they phagocytose intravenously administered dextrans that pass the endothelial barrier, contributing to vascular integrity [126]. Interestingly, in the area postrema, PvMΦ did only allow the passage of small molecules (300 Da) into the brain, suggesting that they restrict the entry of molecules within a defined size range. The barrier supporting properties of PVMΦ seem to be focal, potentially in specific brain areas, because global depletion of PvMΦ with liposomal clodronate did not affect vascular integrity of the BBB in the whole brain [107]. PvMΦ may promote clearance of metabolic waste products and lipid depositions which accumulate in aging animals. Characteristic round inclusion bodies within PvMΦ enlarge with age or in animals on lipid-rich diets and undergo morphological changes, including swollen cell somata, implicating PvMΦ in age-related pathology [78]. Taken together, these and other findings on different brain regions suggest a general phagocytic capacity and scavenger function of PvMΦs and a tight interaction with various components of the neurovascular unit [63, 77, 126].

Given their phagocytic capacity and their location in the perivascular space, PvMΦ may be involved in neurodegenerative diseases like AD, CAA, or cerebral small vessel diseases (SVD), which display protein depositions around the vasculature and vascular dysfunction. Accordingly, depletion of PvMΦ increased CAA severity and the accumulation of APOE in CSF, while its effect on parenchymal plaque load is controversial, with one study reporting a reduction and another an increase [22, 53]. A more detailed discussion of PvMΦ in CAA can be found in the accompanying review by Wu, Bogale, and colleagues [129]. In CADASIL patients, perivascular cells, likely PvMΦ, phagocytose, the granular osmophilic material, is unique in the disease [132]. In mouse models of AD, PvMΦ play a critical role in removing vascular Aβ [53]. In recent studies, it has been demonstrated that anti-Aβ immunotherapy results in formation of immune complexes within vascular amyloid deposits. These complexes can trigger the activation of CD169-positive PvMΦ, which in turn amplify CAA-related vascular permeability, the leakage of plasma proteins, and the infiltration of immune cells linked to microhaemorrhages [118].

One histological characteristic of old brains is the presence of corpora amylacea, which have recently been referred to as wasteosomes [100]. Corpora amylacea are mainly spherical bodies rich in polysaccharides and waste products, with a diameter ranging from < 2 µm to around 20 μm, found in the human brain, especially of the elderly. They are rarely reported in brains of old rodents [11]. J.E. Purkinje and R. Virchow observed these structures in the nineteenth century [11]; however, they received little attention due to lack of a clear connection with neurological disorders. Newly emerging evidence, which demonstrates the release of corpora amylacea from brain tissue into the CSF and the cervical lymph nodes, suggests their role as carriers of waste products out of the brain [99]. In vitro, macrophages expressing the mannose receptor CD206 (Mrc1), which include PvMΦ, can phagocytose these bodies [99]. Additional research will be required to explore whether PvMΦ, along with CD206-positive MnMΦ, can engulf corpora amylacea and whether impairment of this function could result in the build-up of corpora amylacea in the brain. Overall, PvMΦ phagocytosis of corpora amylacea and potentially also remodelling of the ECM could have implications for BBB function, as well as the movement of fluids, molecules, and cells surrounding the brain vasculature, in particular in neuropathological conditions.

## Microglia and perivascular macrophages (PvMΦ) control BBB permeability and neuroinflammation

Microglia and PvMΦ are early responders to pathological insults to the CNS. In young healthy adults, microglia do not seem to influence the BBB tightness, because depletion of CX3CR1-positive microglia (and CAMs) did not affect BBB permeability [91]. However, in 20-month old aged mice, microglia were activated and their depletion (along with the depletion of CAMs) with the CSF1R inhibitor PLX5622 resulted in a mild disruption of BBB function [46]. This vasoprotective effect of microglia was even stronger in young or old mice exposed to mild chronic hypoxia [46, 47]. Microglia depletion aggravated the hypoxia-induced fibrinogen leakage into the brain parenchyma. Similarly, in a model of cerebrovascular injury caused by transcranial ultrasound microglia initially protected the BBB, while the later infiltration of monocyte-derived cells damaged the barrier [73]. By contrast, in a model of inflammatory demyelination, microglia depletion with CSF1R inhibition protected against BBB impairment [135]. Combined, these studies suggest different roles for microglia–endothelial crosstalk in aged animals versus inflammatory/pathological condition and the need for more differentiated research on this topic.

Brain endothelial cells are in general separated from microglia by several barrier structures that include the underlying endothelial basement membrane and the parenchymal basement membrane which together with the attached layer of astrocyte endfeet constitutes the glia limitans. Microglia and PvMΦ primed by inflammatory stimuli therefore mainly communicate with endothelial cells via release of pro-inflammatory cytokines, chemokines, ROS and growth factors, or via extracellular vesicles (Fig. 2) [94, 130, 137]. In a co-culture of LPS-activated microglia and endothelial cells, microglial NADPH activity and TNF secretion altered endothelial junctions containing ZO-1 and occludin with a consequent increase in endothelial monolayer permeability [115]. After systemic injection, LPS acts on brain endothelial cells resulting in increased microglial clustering around CNS blood vessels and microglial contacts with endothelial cells through CCL5-CCR5 signalling (Fig. 2) [51]. After low doses of systemic LPS, microglia were found to maintain the BBB integrity initially, possibly by the ability of microglia to express claudin-5 and form tight junctions with endothelial cells. This may contribute to the preconditioning effect of repeated LPS-injections that protected mice from neuronal and vascular damage in a model of ischemic spinal cord injury [37]. The interaction of endothelial cells and microglia is driven by interleukin-1 (IL-1) production in microglia acting on the receptor IL1R1 in endothelial cells [37, 84]. Together the data show that systemic inflammation induces alterations in myeloid cells near the vasculature, which support endothelial cells and have the capacity to limit barrier leakage.

After initially maintaining the BBB tightness, microglial activation has been reported to disrupt the barrier during systemic inflammation [51]. Although its mode of action is still largely unclear, minocycline, a generally acting antibiotic but presumably also an inhibitor of microglia and MMP-9 activation [45], enables a selective intervention. Minocycline has been shown to improve the compromised BBB function in response to hypoxia in aged mice [46], while microglia depletion with PLX5622 aggravated BBB dysfunction, suggesting a selective effect of minocycline on microglia overactivation or additional effects on other cells. In experimental stroke, minocycline reduced microglial secretion of IL-1β and nitric oxide (NO), resulting in a smaller infarct size [134]. So far, clinical trials using minocycline in stroke, mild AD, and CAA failed to achieve consistent beneficial results [64], but other trials are still ongoing [54]. Overall, a large body of work connects microglia phenotype and BBB state in the pathological context, and it will be a future challenge to use the acquired knowledge and strengthen the beneficial communication lanes between these two cell types.

The proximity of PvMΦ to arteriolar endothelial cells may be relevant for chronic hypertension as suggested in an angiotensin II (Ang II)-infusion murine model. Activation of endothelial angiotensin AT1a receptors correlated with barrier changes in arterioles, allowing Ang II to penetrate through the different layers of the vessel wall and stimulate reactive oxygen species (ROS) production in PvMΦ (Fig. 2). This led to a more severe BBB disruption as shown by the protective effect of clodronate-mediated PvMΦ depletion in Ang II-treated chronic hypertensive mice [107], although the depletion by PLX5622 of both PvMΦ and microglia did not protect the barrier in another study of chronic hypertension [62]. In this disease context, the interaction of endothelial cells and PvMΦ impaired the BBB and resulted in vascular dysfunction, leading to cognitive deficits in chronic hypertension [31]. Other studies in experimental subarachnoid haemorrhage showed that intracerebroventricular injected clodronate liposomes depleted PvMΦ and delayed blood clearance but had a beneficial effect on neuronal cell death, neuroinflammation, neurological impairment, and microvasospasms [68, 123]. These studies show that PvMΦ effects on BBB function are variable, which may be related to either the experimental set-ups/model investigated or potentially the heterogeneity of PvMΦ in different locations, at different developmental stages or under different metabolic conditions.

In addition to ROS, VEGF has been implicated in paracrine signalling between PvMΦ and endothelial cells. In mice fed with a high-fat diet, the glucose transporter GLUT1 in brain endothelial cells and the supply of glucose to the brain transiently decreased, apparently stimulating VEGF expression in PvMΦ [57]. By releasing VEGF, PvMΦ rescued low levels of endothelial GLUT1 and partially ameliorated diet-induced cognitive impairment in diet-induced obesity (Fig. 2) [57]. In contrast to its protective effects, high levels of VEGF also increase vascular permeability during hypoxia [110]. Depletion of CAMs by clodronate liposomes in an MCAO model of stroke lowered VEGF-A expression and improved the neurological deficit and cortical vascular leakage in the first hours after stroke [92]. Importantly, the latter study did not exclude a vasculoprotective effect of PvMΦ during the chronic phases of pathology.

## Microglia as janitors of the vascular environment

Microglia have a large phagocytic potential and are instrumental in removing excess neuronal synapses, protein accumulations, debris depositions, and dead cells. Through their unique location, vessel-associated microglia are presented with accumulations derived both from the circulation and the CNS parenchyma, which can affect microglia function and their effects on vascular pathology.

**Fig. 3:**
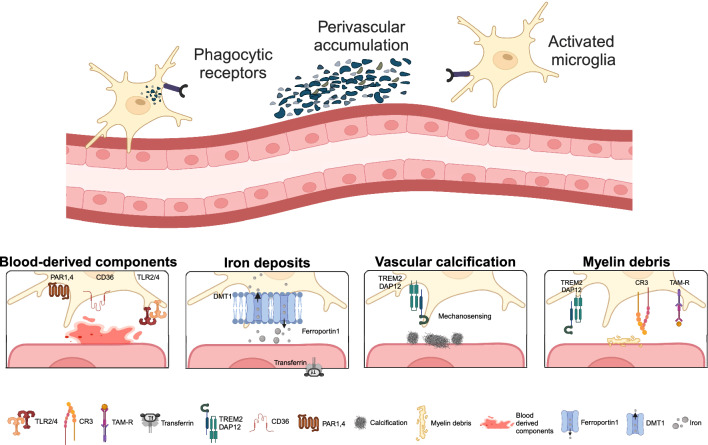
Scheme summarizing macrophage effects on perivascular deposits. PvMΦ and microglia remove blood-derived components, iron deposits, calcifications, and myelin debris. Information on vascular amyloid deposits is provided in the accompanying review by Wu, Bogale, and colleagues [129]. ABCA1, ATP-binding cassette transporter A1. CR3, complement receptor 3. CD36, cluster of differentiation 36. DAP12, DNAX activation protein 12. DMT1, divalent metal transporter 1. PAR1, 4, protease activated receptor 1, 4. TLR2/4, Toll-like receptor 2/4. TREM2, triggering receptor expressed on myeloid cells 2

Perivascular deposits of iron occur commonly in the human brain and increase with age and in neurodegenerative diseases, such as CAA, Parkinson’s disease (PD), and MS [19]. In such disorders, microglia accumulate iron (Fig. 3). Recent work on human iPSC-derived microglia suggests that iron loading reduces microglial phagocytic activity, enhancing their immune response profile during inflammation [61]. Finally, ferroptosis, iron-mediated cell death, and concomitant generation of ROS are predicted to affect microglia and nearby vascular cells, potentially affecting BBB functionality [105].

Like iron deposits, and often co-occurring with it, calcifications are found in different layers of cerebral vessels. They are associated with vascular aging, movement disorders like primary familial brain calcification, and possibly other neurodegenerative diseases. Patients with mutations in microglia genes like CSF1R, required for microglial development and survival [25], harbour intracerebral calcifications in the white matter and other brain areas, highlighting the importance of microglia in controlling these deposits [44]. Microglia have been proposed to deal with vascular calcification at multiple levels including regulating the mineralization and proteostasis of the ECM or phagocytosing calcium phosphate precipitates [136]. Calcifications can be sensed via ligand-receptor signalling involving TREM2, a key regulator of microglia function, or through increased vessel stiffness, resulting in a distinct microglia activation profile (Fig. 3) [136]. In a mouse model for primary familial brain calcification, both microglia (and CAM) depletion by PLX5622 treatment and TREM2 deficiency exacerbated vascular calcifications, the latter of which may be related to microglial exhaustion and loss of phagocytic efficiency [136]. Consequently, effective removal of calcifications by microglia is considered beneficial for vascular and overall brain health.

When vascular integrity is disrupted in intracerebral haemorrhage, microglia migrate to the site of bleeding and phagocytose erythrocytes [117]. Blood-derived proteins, including, prothrombin/thrombin, fibrinogen/fibrin, and others, activate brain macrophages (Fig. 3). Erythrocyte phagocytosis contributes to the resolution of inflammation [12, 117]. However, when stimulated by fibrin, microglia activation via CD11b/CD18 can also be responsible for axonal damage and oxidative stress, further affecting the immediate microenvironment and phagocytic activity of microglia [21]. It is still unclear whether microglia lose their efficiency in clearing (micro-)haemorrhages over time or whether the initiated microglial activation program overrules the clearing effects. Currently, the potential of blood protein-targeting immunotherapy is being explored as a promising strategy for the treatment of neuroinflammatory and neurodegenerative diseases. Studies using a monoclonal antibody against a fibrin epitope have already yielded promising effects [106].

Neuroinflammation and degeneration cause demyelination and myelin debris, a known trigger for microglia phagocytosis. While a plethora of data on microglial myelin uptake exists, evidence for myelin debris accumulation and processing near the vasculature is rare [33]. Clearing perivascular myelin debris seems imperative for remyelination, as debris hampers neurite growth and differentiation of oligodendrocyte precursors, which migrate along the vasculature to the site of remyelination [85]. Recent literature further revealed myelin engulfment by the endothelial cells in models of spinal cord injury and MS [128]. However, this process stimulated endothelial-to-mesenchymal transition, a process involved in BBB dysfunction, macrophage recruitment and fibrotic scar formation, critically questioning the role of endothelial cells as professional phagocytes [140].

Overall, the phagocytic activity of microglia is essential for brain health, especially near the vasculature, where extracellular deposits and protein aggregates co-exist. Even though many phagocytic receptors are polymodal, some extracellular deposits, like vascular calcification or Aβ, elicit a specific microglia signature, differing slightly from a general phagocytic profile. It is challenging to distinguish between effects related to excessive accumulation or age-related microglia exhaustion, as they co-occur in neurodegenerative diseases and result in reduced microglial phagocytic capacity. Future investigation of the phenotype and function of vessel-associated microglia versus their parenchymal counterpart may aid in identifying microglial populations with defined functions.

### Antigen presentation by endothelial cells and macrophages

Macrophages and endothelial cells are involved in antigen-presentation, which is a prerequisite for activation and proliferation of T cells to form protective T-cell responses against danger or pathogen-associated signals. Antigen-presenting cells (APCs) engulf and process antigens and re-express the processed antigen on their cell surface in association with major histocompatibility (MHC) class I molecules to CD8 T cells or with MHC class II molecules to CD4 T cells. In addition to the interaction of the T-cell receptor with the peptide-MHC complex, activation of naïve T cells requires a second signal from the APC provided by engagement of co-stimulatory molecules followed by a third signal through instructive APC-secreted cytokines. The ability to deliver these three signals is a characteristic of “professional” APCs [4] which are, thereby, equipped to initiate a primary T-cell-dependent immune response. Activation of T cells that have previously been exposed to their cognate antigen is, however, less dependent on additional co-stimulatory signals. “Non-professional” APCs, which lack expression of co-stimulatory signals, may therefore be able to stimulate secondary T-cell-mediated immune responses by presenting antigen to previously primed T cells.

In the CNS, constitutive expression of MHC class I and II molecules including on the tissue-resident microglial cells and CAMs is very low or absent. However, in neuroinflammation expression of MHC molecules is upregulated in CNS resident cells [summarized in 82]. Upregulation of MHC class II and co-stimulatory molecules has mainly been observed on microglial cells in virtually all inflammatory conditions [summarized by 6]. However, although microglial cells can phagocytose myelin, suggesting that they could activate myelin-specific CD4 T cells, when cultured in vitro MHC class II expressing microglial cells induced apoptosis of CD4 T cells rather than promoting T-cell proliferation [34]. This suggests that antigen presentation by microglial cells may dampen neuroinflammation.

Prior to our current understanding of the molecular mechanisms mediating the multi-step extravasation of T cells across the BBB, antigen presentation by brain endothelial cells was considered to initiate CD4 T-cell entry into the CNS in the context of MS and EAE. Developing methodology to purify and grow primary brain microvascular endothelial cells (BMECs) as in vitro models of the BBB allowed in the early 1990s to show that BMECs do not express MHC class II but its expression can readily be induced by stimulation with interferon-γ (IFN- γ) [79, 102, 111]. These early in vitro studies also showed that MHC class II expressing BMECs failed to induce T-cell proliferation. Rather, BMECs exposed to myelin-specific CD4 T cells but also co-incubated with CD4 T cells recognizing non-myelin antigens were eventually lysed by these CD4 T cells in an antigen-dependent and MHC class II-restricted manner [79, 102, 111]. These early studies thus already showed that, at least in vitro*,* BMECs can present protein antigens on MHC class II molecules to CD4 T cells but lack the co-stimulatory cues to trigger T-cell proliferation. A recent study employing human hCMEC/D3 as an in vitro BBB cell line model showed that these BMECs can internalize myelin, target the myelin to the endo-lysosomal compartment and present myelin/MHC class II complexes on their surface, where they stimulate antigen-dependent CD4 T-cell migration across the hCMEC/D3 monolayer [70]. Although these data suggest that MHC class II-restricted antigen presentation by BMECs can contribute to autoimmune neuroinflammation by enhancing CD4 T-cell recruitment into the CNS and several studies localized MHC class II to BMECs in brain or spinal cord sections from rodents suffering from EAE [113], there are no data that causally links MHC class II expression at the BBB to MS pathogenesis. While the expression of MCHII on brain endothelial cells is a much-debated topic, MHC class II expression by PvMΦ has also been suggested by in vivo immunostaining studies [58, 122].

A functional contribution of MHC class II-mediated antigen presentation by MnMΦ in the re-activation of CD4 T cells has been postulated in EAE. MnMΦ are localized in a strategic position in the CSF draining subarachnoid space, making them ideal candidates to take up CNS antigens from the CSF and to present them to T cells patrolling the CNS border compartments during immune surveillance but also in neuroinflammation. Elegant two-photon in vivo imaging studies in rat EAE showed that myelin-specific CD4 T cells form immunological synapses with local myeloid cells in the leptomeninges that could be either MnMΦ or dendritic cells. Subsequently, these contacts led to NFAT translocation into the T-cell nucleus and T-cell activation as a prerequisite for the subsequent infiltration of these encephalitogenic CD4 T cells into the CNS parenchyma [5, 95]. By contrast, ovalbumin-specific CD4 T cells did not form stable interactions with local myeloid cells, unless ovalbumin was injected into the CSF and was taken up by local myeloid cells. While these observations suggest that antigen presentation by local APCs could play a role in re-activation of CD4 T cells, the identity of the APCs is still unclear. Importantly, specific ablation of MHC class II in microglia and CAMs failed to affect CD4 T-cell-mediated EAE. Only genetic deletion of MHC class II in dendritic cells and monocyte-derived cells protected mice from developing clinical EAE [60]. Taken together, these observations suggest that MHC class II-mediated antigen presentation by peripheral professional APCs such as dendritic cells, rather than brain endothelial cells or brain macrophages, is critical for autoimmune neuroinflammation.

In contrast to MHC class II, there is vast in vitro and in vivo evidence for a role of brain endothelial MHC class I-dependent antigen presentation in the pathogenesis of cerebral malaria. In a mouse model of cerebral malaria, parasite-specific CD8 T cells elicit CNS pathology, probably by attaching to CNS microvessels and killing brain endothelial cells that have internalized plasmodium antigen [98]. Importantly, post-mortem human brain samples confirm accumulation of CD8 T cells in CNS microvessels [101]. In vitro studies have supported this concept as it was shown that IFNγ stimulated primary mouse BMECs can phagocytose parasites and cross-present plasmodium antigens on MHC class I molecules to CD8 T cells, leading to CD8 T-cell-mediated killing of the BMECs [55]. A recent study employing an elegant genetic mouse model that allows endothelial-specific ablation of individual MHC class I molecules provided the first direct in vivo evidence for the involvement of MHC class I-restricted antigen presentation on BMECs in the pathogenesis of experimental cerebral malaria [28]. These observations underscore the importance of CD8 T-cell interactions with different MHC class I-peptide complexes on brain endothelium in vivo*,* triggering different CD8 T-cell responses, including CD8-T-cell-mediated BBB breakdown. In fact, adoptive transfer of antigen-specific CD8 T cells into transgenic mice expressing the respective antigen in brain endothelial cells suffices to induce CD8 T-cell-mediated apoptosis of BMECs in vivo*.* The endothelial cell death leads to microvascular damage resembling the pathology observed in Susac syndrome, a neuroinflammatory disorder with CNS endotheliopathy [43]. These observations, therefore, suggest that Susac syndrome is a CD8 T-cell driven endotheliopathy against unidentified antigens presented on MHC class I by brain endothelial cells.

Since BBB endothelial cells are highly polarized, it cannot be taken for granted that they can also take up antigens from the abluminal and thus CNS facing side. However, two independent studies showed that BMECs can take up exogenous antigen from their abluminal side in vitro and in vivo*,* process and cross-present it on their luminal side in an MHC class I-dependent manner to antigen-specific CD8 T cells [3, 28]. In vitro studies suggested that inflamed BMECs triggered full activation of naïve CD8 T cells leading to their proliferation and differentiation to cytotoxic effector CD8 T cells [3]. Thus, either BMECs possess the entire machinery required for cross-presentation of antigen on MHC class I molecules or MHC class I-peptide complexes on BMECs may suffice to activate naïve CD8 T cells as previously shown for naïve CD8 T-cell activation in APC-free systems [41, 89].

Recognition of their cognate antigen on MHC class I molecules on the BMEC surface was proposed to facilitate CD8 T-cell entry into the CNS [38]. This would require firm interaction of the T-cell receptor with peptide-MHC class I complexes on BMECs under physiological flow. Investigating CD8 T-cell interaction with BMECs in the absence and presence of their cognate antigen and/or functional expression of MHC class I by in vitro live cell imaging showed no involvement of endothelial antigen presentation in mediating the initial arrest of CD8 T cells on BMECs under physiological flow [3, 42]. Rather, MHC class I-restricted antigen presentation influenced post-arrest behaviour of CD8 T cells on BMECs by abrogating their crawling and subsequent diapedesis, leading to CD8 T-cell-mediated apoptosis of BMECs [3, 42]. Thus, engagement of the T-cell receptor with the MHC class I-peptide complexes on BMECs rather induces a stop signal for T cells on BMECs hindering their mobility on adhesion molecules and favouring their focal cytotoxic activity [26]. In vivo imaging in both a mouse model of cerebral malaria and in mice expressing ovalbumin as a model antigen in oligodendrocytes showed a significant reduction in the crawling speed of CD8 T cells in CNS microvessels when endothelial MHC class I and the cognate CD8 T-cell antigen were present, supporting the notion that brain endothelial MHC class I-dependent cross-presentation of luminal and CNS-derived antigen reduced motility of CD8 T cells in CNS microvessels in vivo [3, 28].

Interestingly, focal BBB breakdown, as observed in CD8 T-cell-mediated autoimmune neuroinflammation in vivo*,* was found to be independent of the cytotoxic activity of CD8 T cells. In mice expressing the antigen ovalbumin in oligodendrocytes, CAMs were very efficient in engulfing ovalbumin, suggesting that there is only limited antigen uptake and presentation by BMECs in vivo which suffices to reduce CD8 T-cell crawling speed within CNS microvessels but not necessarily CD8 T-cell-mediated focal BBB breakdown [3]. This suggests that as long as CAMs efficiently engulf CNS-derived antigens, uptake of antigens by BMECs may be limited, leading to only low densities of antigen-MHC-class I complexes on the BBB that may reduce CD8 T-cell crawling speeds but not suffice to trigger CD8 T-cell-mediated BBB breakdown. This hypothesis is in accordance with the observation that targeted expression of a neo-antigen in BMECs result in haemorrhagic BBB breakdown upon transfer of neo-antigen-specific effector CD8 T cells [43]. Thus, the nature and severity of CNS tissue damage as well as the involvement of CAMs and microglial cells in antigen presentation influence the availability of antigens to be taken up and processed by BMECs and antigen-specific interactions with CD4 and CD8 T cells in CNS microvessels.

## Conclusion

In the CNS, macrophages and endothelial cells act as allies in multiple biological processes. Their interactions are evident from early development to old age. Focusing on the adult brain in health and disease, the present review aims to outline that considering the diversity of the two cell groups helps understand some seemingly contradictory observations. In this respect, there has been tremendous progress in recent years. Nevertheless, many studies rely on end-point investigations and provide a rather static picture. We propose that intravital investigations that take dynamic changes in the endothelial–macrophage interaction into account may dissolve some apparent discrepancies in the future. Even with the current state of knowledge novel therapeutic interventions should target both key players.
